# Brachytherapy in Brain Metastasis Treatment: A Scoping Review of Advances in Techniques and Clinical Outcomes

**DOI:** 10.3390/cancers16152723

**Published:** 2024-07-31

**Authors:** Sandra Leskinen, Netanel Ben-Shalom, Jason Ellis, David Langer, John A. Boockvar, Randy S. D’Amico, A. Gabriella Wernicke

**Affiliations:** 1 Downstate Medical Center, State University of New York, New York, NY 11203, USA; sandra.leskinen@downstate.edu; 2 Department of Neurological Surgery, Zucker School of Medicine at Hofstra/Northwell, Lenox Hill Hospital, New York, NY 10075, USA; 3 Department of Radiation Medicine, Zucker School of Medicine at Hofstra/Northwell, Lenox Hill Hospital, New York, NY 10075, USA

**Keywords:** radiotherapy, radiation, Cesium-131, Iodine-125, neuro-oncology

## Abstract

**Simple Summary:**

Brain metastases are cancerous growths that spread to the brain from other parts of the body, causing severe health problems. This review explores brachytherapy, a treatment where radioactive sources are placed directly into or near tumors or tumor beds to deliver targeted radiation, with the goal of minimizing damage to healthy brain tissue. Here, we aim to summarize the history and recent advancements of brachytherapy techniques and clinical outcomes in brain metastasis treatment, showcasing its benefits and limitations and providing a clearer understanding of how brachytherapy may potentially improve quality of life for patients with brain metastases.

**Abstract:**

Brain metastases pose a significant therapeutic challenge in the field of oncology, necessitating treatments that effectively control disease progression while preserving neurological and cognitive functions. Among various interventions, brachytherapy, which involves the direct placement of radioactive sources into or near tumors or into the resected cavity, can play an important role in treatment. Current literature describes brachytherapy’s capacity to deliver targeted, high-dose radiation while minimizing damage to adjacent healthy tissues—a crucial consideration in the choice of treatment modality. Furthermore, advancements in implantation techniques as well as in the development of different isotopes have expanded its efficacy and safety profile. This review delineates the contemporary applications of brachytherapy in managing brain metastases, examining its advantages, constraints, and associated clinical outcomes, and provides a comprehensive understanding of advances in the use of brachytherapy for brain metastasis treatment, with implications for improved patient outcomes and enhanced quality of life.

## 1. Introduction

Brain metastases are secondary tumors that have spread to the brain from primary cancer sites elsewhere in the body. They are a significant cause of morbidity and mortality in cancer patients and represent one of the most common neurological complications associated with systemic disease [1]. Whole-brain radiation therapy (WBRT) was once the standard radiotherapeutic approach to brain metastases (BMs) [2]. While effective at disease control, WBRT is also prone to iatrogenic injury of at-risk healthy organs in the brain like the hippocampus, leading to post-irradiation cognitive impairment and worsened quality of life. With the intent to minimize the collateral effects of radiation on healthy brain tissue, new radiation protocols have given rise to more targeted techniques such as stereotactic radiosurgery (SRS) and hippocampal avoidance WBRT [3,4]. Importantly, SRS is now the standard of care in most cases of BM [5].

Brachytherapy involves the placement of radioactive sources directly into, or adjacent to tumor sites or resection cavities. This method allows for highly localized radiation to be administered to tumor tissues or to eradicate microscopic disease post-tumor resection, while sparing surrounding healthy structures [6]. Primarily utilized for conditions like prostate and cervical cancers, its applications have extended to include tumors of the central nervous system like BM [7]. The expansion of its applications is supported by technological improvements, a better understanding of tumor radiobiology, and a need for therapies with reduced long-term side effects.

This literature review will explore current applications of brachytherapy in the treatment of brain metastases, showcasing its advantages, limitations, and clinical outcomes. We would like to emphasize that this review is not intended as a clinical guideline or a definitive clinical reference. Instead, it is meant to provide a comprehensive overview of the current state of brachytherapy used in the treatment of BM, summarizing recent advancements and clinical outcomes. This review aims to inform both researchers and clinicians about the evolving landscape of brachytherapy and its potential future applications to brain metastases.

## 2. Methods

Adhering to PRISMA Extension for Scoping Reviews guidelines (PRISMA-ScR), a comprehensive literature search was conducted using two online databases: PubMed/MEDLINE and Embase. ClinicalTrials.gov was similarly queried to determine the number of ongoing brachytherapy trials for BM treatment. This review was not prospectively registered.

The following search was performed: (“brachytherapy”) AND (“brain metastasis” OR “brain metastases”). Titles and abstracts of the resulting articles were screened by one reviewer (SL). English language in vivo prospective studies, retrospective studies, randomized controlled trials, and case series/case studies with mention of brachytherapy and BM in the title and/or abstract were further scrutinized for eligibility. Non-English language studies, ex vivo studies, in vitro studies, feasibility studies, planning/modeling studies, quality control studies, pediatric studies, editorials, commentaries, and abstract-only articles were excluded. The abstracts of screened studies were further analyzed and included in the final analysis if they reported brachytherapy techniques, isotopes, and patient outcomes. The final search was conducted on 19 July 2023.

## 3. Results

**Figure 1:**
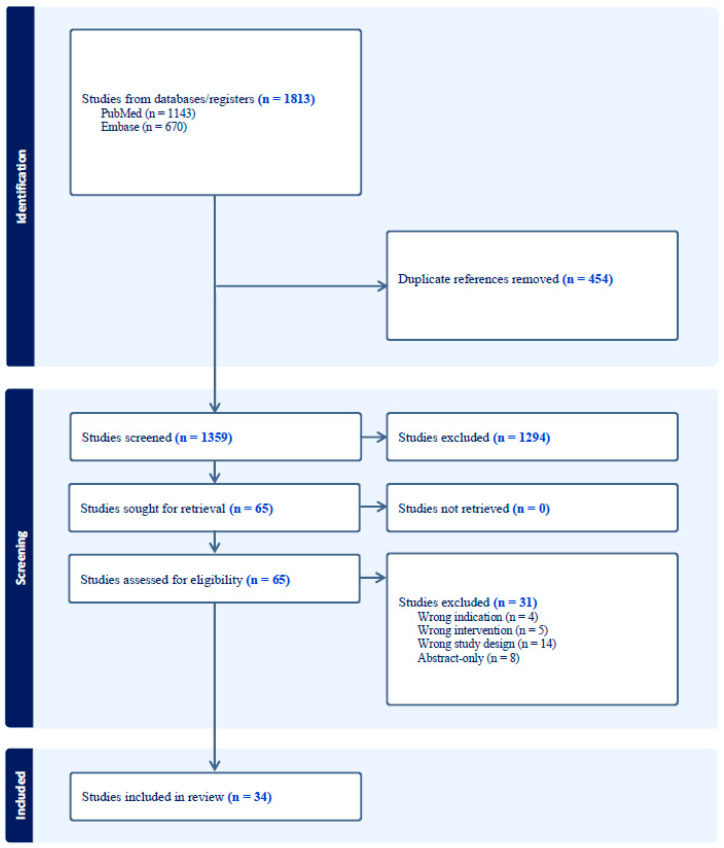
PRISMA flowchart detailing the search strategy and exclusion criteria used in this review.

Our initial search yielded 1813 articles. Following the removal of duplicates, 1359 articles were screened. Of these, 65 articles were further assessed for eligibility (Figure 1). Ultimately, 34 studies met inclusion criteria reporting on brachytherapy techniques in BM treatment, the isotopes utilized, and patient outcomes (Table 1 and Table 2). In addition, we identified three registered national clinical trials examining the use of Cs-131 brachytherapy for BM treatment (Table 3).

Among the 34 included studies were 4 case reports, 21 retrospective cohort studies, 4 prospective cohort studies, and 5 phase I/II clinical trials—4 of which evaluated the same patient cohort. Studies primarily reported the outcomes of patients treated in the USA (79.4%) and Germany (14.7%). Primary cancer types included lung (88.2%), breast (76.5%), melanoma (61.8%), renal (47.1%), gynecological (38.2%), colorectal (32.4%), gastrointestinal (20.6%), esophageal (11.8%), prostate (8.8%), thyroid (8.8%), and other rarer types like hepatobiliary, bladder, and tonsillar (20.6%).

The number of patients with BM treated with brachytherapy ranged from 1 to 95 across studies, with a total of 1135 patients. Twenty-five (73.5%) studies assessed brachytherapy use in newly diagnosed or untreated BM (Table 1), seven (20.6%) studies evaluated recurrent BM (Table 2), and two (5.9%) evaluated both newly diagnosed and recurrent. Twenty-one studies reported on single BM—both newly diagnosed and recurrent—and eighteen studies assessed brachytherapy use in single newly diagnosed BM. Iodine-125 (I-125) seeds were utilized as the radioactive source in 14 studies, I-125 GliaSite in 1 study, Cesium-131 (Cs-131) seeds in 13 studies, Cs-131 GammaTile in 4 studies, and both I-125 and Cs-131 seeds in 2 studies.

A. Radioactive Isotopes: Iodine-125

Of the 17 total studies assessing I-125 brachytherapy, 14 were retrospective cohort studies, 2 were prospective cohort studies, and 1 was a case report. Fourteen studies utilized I-125 seeds as the radioactive source, one utilized GliaSite, and two studies used both I-125 and Cs-131 seeds, treating a total of 792 patients with BM.

I-125 has an extensive history [41,42,43]. In 1981, Gutin et al. placed both permanent and temporary radioactive sources made of I-125, gold-198, and iridium-192 directly into the tumors of 37 patients with recurrent primary or metastatic brain tumors [44]. While all the isotopes were effective to some degree, I-125 in particular became favored for its low energy emissions [44]. In 1989, Prados et al. published a retrospective study on the use of interstitial brachytherapy in the treatment of patients with single metastatic lesions to the brain. I-125 radioactive sources were placed directly into the lesion. Ten out of fourteen patients survived for over one year, eight showed stabilization in neurological function, and six showed improvement on follow-up imaging [45].

The largest studies evaluating the efficacy and safety of I-125 brachytherapy in the treatment of BMs were conducted following surgical resection and permanent I-125 seed implantation to treat patients with newly diagnosed BM [19]. These studies revealed high local control rates and median survival times with low incidences of radiation necrosis (RN). In one study, the average radiation dose delivered was 150 Gy with a seed activity ranging from 4.04 to 40.38 mCi. The authors reported a 93% local control rate and a median overall survival of 14 months. All patients experienced stable or improved Karnofsky performance status (KPS) scores post-treatment and the reported RN rate was 6% [19].

In a retrospective study of 219 patients with single, newly diagnosed BMs who were either treated with SRS (*n* = 142) or permanent interstitial I-125 brachytherapy, Ruge, Kocher et al. reported a local control rate of 94.6% and a median overall survival of 8 months in patients treated with brachytherapy [24]. In another 2011 study, Ruge, Suchorska et al. retrospectively reviewed 90 patients with single BMs who were treated with stereotactic I-125 brachytherapy. The cumulative surface dose to the BMs was 50 Gy. They found that median overall survival was 8.5 months, and the 1-year local control rate was 94.6% [25]. Ostertag et al. evaluated 93 patients who were treated for single BMs with interstitial I-125 brachytherapy with a reference tumor dose of 60 Gy. In patients with newly diagnosed BM treated with I-125 brachytherapy only, median overall survival was 15 months. In patients with previously treated BMs, median overall survival was 6 months. 79% of patients had a stable or improved KPS score at three months. No patients developed RN requiring surgical intervention [18].

**Figure 2:**
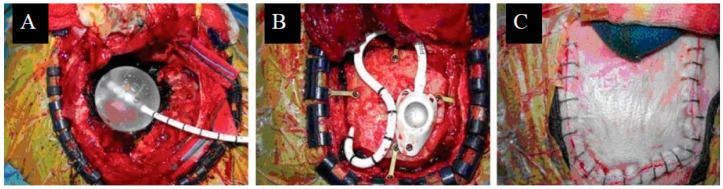
Treatment with GliaSite: (**A**) GliaSite balloon placement within resection cavity. (**B**) Reservoir placement. (**C**) Scalp closure following GliaSite placement. Originally published in Wernicke et al. [48] Licensed under the Creative Commons Attribution-Noncommercial-Share Alike 3.0 Unreported License.

While many studies evaluated permanent forms of I-125 brachytherapy, temporary forms, such as that delivered by the GliaSite radiation therapy system (Figure 2), similarly represented a unique approach to treating BM following surgical resection. Though no longer in use, GliaSite was an FDA-approved balloon catheter device that was placed in the resection cavity immediately after tumor removal. After surgical placement, the balloon is filled with an aqueous form of a radioactive isotope, typically I-125, which delivers a high dose of radiation to the tumor bed while sparing the surrounding normal brain tissue [46]. The aqueous form of the isotope provides a conformal dose distribution to the resection cavity, allowing for controlled radiation delivery. The treatment duration with GliaSite typically ranges from a few days to a week, after which the radioactive solution is withdrawn, and the balloon is removed [47]. Reportedly, GliaSite therapy post-resection was able to achieve local control rates comparable to those achieved with WBRT or SRS, but also exhibited a high rate of RN, with a 1-year actuarial incidence rate of 23% [22]. Although I-125 brachytherapy was found to be effective in BM treatment with local control rates and median overall survival paralleling SRS and WBRT, particularly when coupled with surgical resection, the rates of RN were concerning [14,19].

B. Radioactive Isotopes: Cesium-131

Of the 19 total studies assessing Cs-131 brachytherapy, 9 were retrospective cohort studies, 2 were prospective cohort studies, 5 were phase I/II clinical trials, and 3 were case reports. Thirteen studies utilized Cs-131 seeds as the radioactive source, four utilized GammaTile, and two studies used both I-125 and Cs-131 seeds, treating a total of 466 patients with BM.

In a phase I/II clinical trial of 24 patients with BMs, Wernicke et al. implanted permanent intracavitary Cs-131 stranded seeds with a median seed activity of 3.82 mCi and a prescribed dose of 80 Gy 5 mm from the surface of the resection cavity. Local freedom from progression (FFP) was 100% and 1-year regional FFP was 93.8% due to one patient experiencing leptomeningeal spread. Median overall survival was 9.9 months and 1-year overall survival was 50%. They reported no instances of RN [28]. Another study described 13 patients with previously irradiated BMs who were treated with Cs-131 stranded seeds with the same dose prescription as in the phase I/II study. Median seed activity was 2.25 mCi and median number of implanted seeds was 19. The 1-year local FFP was 83.3%, median overall survival was 7 months, and 1-year overall survival was 24.7%, with only one reported case of RN [39].

As salvage therapy for recurrent BMs, Cs-131 brachytherapy has shown efficacy, providing good local control with minimal toxicity. A prospective study of 20 patients who had undergone failed treatment of BMs with SRS were treated with resection and Cs-131 seeds embedded into the resection cavity. Two tumors recurred after 1.6 years follow-up, amounting to a 1-year incidence of progression rate of 8.4% [49]. A similar study in patients with BMs that had previously been treated with either SRS or WBRT were treated for recurrence with embedded Cs-131 seeds demonstrated 83.3% 1-year local FFP and a median survival of 7 months [39]. In this study, only one patient experienced RN.

**Figure 3:**
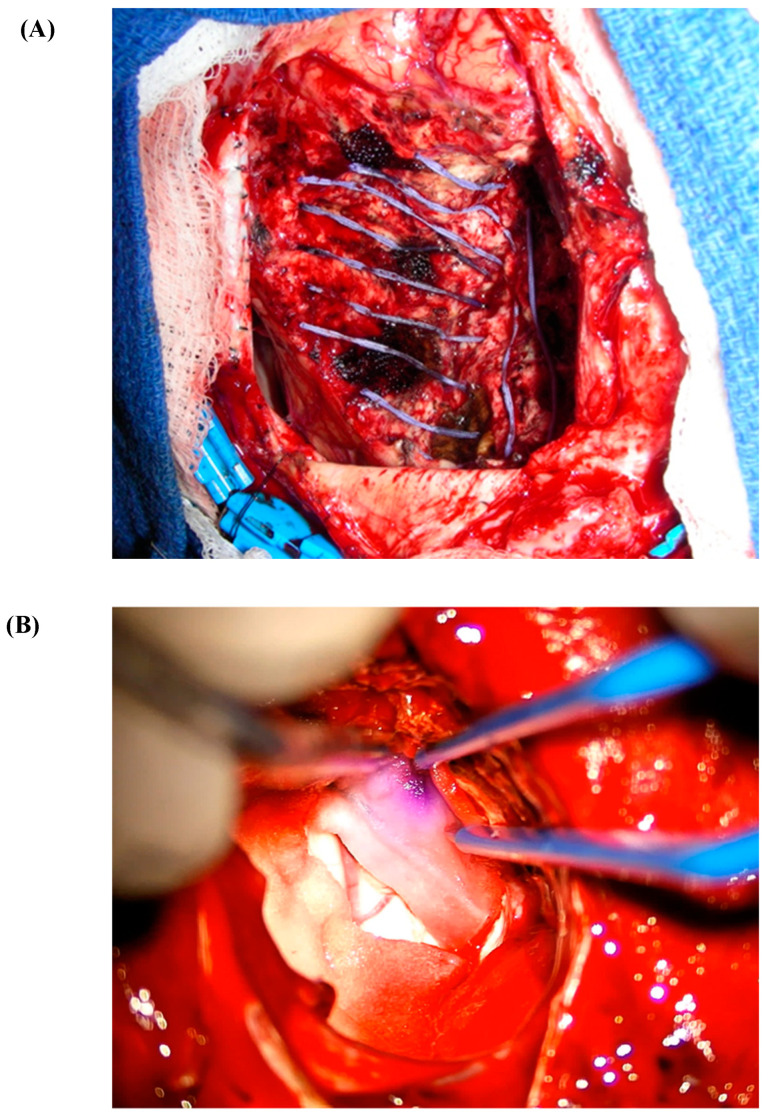
(**A**) Cs-131 stranded seed on a nylon suture. (**B**) Cs-131 seed embedded in collagen (GammaTile).

It is important to note that post-surgical resection cavity shrinkage affects the distribution and effectiveness of brachytherapy. After surgical resection, the tumor cavity shrinks by a median percent volume of 30%, with larger resection cavities exhibiting greater shrinkage. This volume decrease occurs quickly, within 0–3 days post-resection [50]. In the placement of radioactive seeds (Figure 3), post-resection cavity shrinkage brings the seeds closer together, and closer to healthy brain tissue, affecting dose strength and radiation homogeneity, possibly contributing to an increased RN risk [51,52]. In a retrospective study that included patients from their phase I/II trial, Wernicke et al. observed significantly less cavity shrinkage in patients treated with Cs-131 versus SRS during the first-month post-resection [30]. This reduced shrinkage after 1 month coincided with the absorption of 88% of the Cs-131 dose, potentially diminishing the RN risk by minimizing the decrease in inter-seed distance [30].

**Figure 4:**
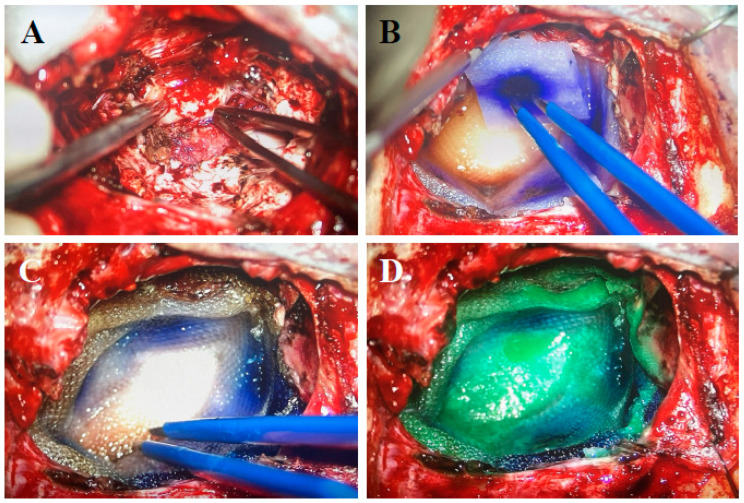
GammaTile placement: (**A**) Resection cavity free of tumor. (**B**) Cavity layered with GammaTiles. (**C**) Placement of Surgicel on top of GammaTiles. (**D**) Bioabsorbable glue.

Dosimetric analyses seem to favor Cs-131 over I-125, revealing that Cs-131 exposed smaller mean volumes of brain tissue to radiation and delivered lower doses to equivalent clinical target volumes compared to I-125. Furthermore, Cs-131 demonstrated higher homogeneity and conformity indices, suggesting more uniform and accurate dose delivery [33]. Additionally, since Cs-131 has a short half-life of 9.7 days and about 90% of the intended dose is absorbed within 1 month following Cs-131 placement, as opposed to 32% with I-125 placement, patients can commence adjuvant systemic therapy as soon as 1 month following resection and seed implantation [53]. However, despite its potential in minimizing RN rates, Cs-131’s relative novelty in BM treatment needs further study to establish long-term outcomes [54]. New techniques like the FDA-approved GammaTile (GT Medical Technologies, Tempe, AZ, USA) have been formulated with the intent of mitigating dosing and safety concerns. In this formulation, radioactive seeds are encapsulated in titanium and embedded in a collagen carrier that can be placed in the tumor resection cavity (Figure 4) [37,55,56]. Studies evaluating this technique are ongoing.

C. Clinical Outcomes and Safety

The ability of brachytherapy to concentrate high radiation doses directly to the tumor site while preserving surrounding normal tissue has been associated with increased local control rates, particularly for patients with one or a few lesions. In their study of 72 patients with BM, Petr et al. reported local control rates that were directly comparable to the control rates seen in WBRT while sparing patients of the often-deleterious neurological consequences post-WBRT [19]. In a retrospective study by Ruge et al. evaluating the efficacy of stereotactic I-125 brachytherapy in non-resected BM, the authors found rates of local control and median overall survival similar to SRS [24]. In a study comparing post-resection I-125 brachytherapy to external beam radiotherapy (EBRT) in BM treatment, Yang et al. found no significant difference in overall survival and progression-free survival between patients treated with EBRT and those treated with I-125 brachytherapy, with the latter group showing superior six-month progression-free survival [32]. As an adjunctive therapy, brachytherapy complements surgical resection by targeting potential microscopic remnants to minimize tumor recurrence [21,29].

Brachytherapy has also shown favorable outcomes in the treatment of large, irregular BMs [54]. In a 2016 retrospective study of 95 patients with BM treated with resection and I-125 permanent seed placement, Raleigh et al. observed crude local control rates as high as 90% and an RN rate of approximately 15% [21]. The risk of RN was higher with lesions that had undergone previous treatment with SRS. They compared this to similar studies of patients with BM who underwent surgical resection plus WBRT and SRS plus WBRT, which reported local control rates of 86% [57] and 82–89% [58,59], respectively. Raleigh et al. concluded that surgical resection plus brachytherapy is comparable to the effectiveness of SRS or WBRT, and that the technique may actually be a better option than SRS in treating large tumors due to their observed local control rate of 90% even with the inclusion of tumors that measured up to 76 cm^3^ [21]. Similarly, in a prospective trial of 42 patients with a total of 46 BMs, Wernicke et al. applied surgical resection and Cs-131 brachytherapy to patients with BMs measuring larger than 3 cm [29]. The authors secured a local FFP rate of 100% and a 1-year regional FFP of 80%. They also reported no cases of RN. In comparison to other studies using SRS that reported local control rates of 94% [60] and 85.5% in tumors greater than 3 cm [61], brachytherapy appears comparable in maintaining local control [29].

Safety studies by Yondorf et al. indicate that standard protective measures are sufficient for handling Cs-131 seeds, ensuring safety for both medical personnel and patient contacts [62]. The study concluded that the use of leaded aprons and gloves in the operating room is adequate to protect surgical staff. Individuals who interact with a treated patient should maintain a conservative distance of 35 cm for about 2 weeks postoperatively due to the relatively short half-life of Cs-131 [62]. In a study that included radiation precautions following the implantation of I-125 sources, radiation levels were measured at a distance of 1 m from the patient. If the radiation levels were at or above 0.2 mR/h, patients were provided with lead shielding in the form of lead sheets inserted into special hats. The radioactive sources were left in place permanently, with their activity naturally decreasing over time. Radiation precautions were necessary for 2 to 4 months until the exposure levels at 1 m fell below 0.2 mR/h [14].

D. Knowledge Gaps and Future Directions

Clear criteria to identify which patients with BMs may benefit from brachytherapy, versus other treatment modalities, are lacking. Currently, the American Brachytherapy Society consensus statement on intraoperative radiation therapy recommends the use of brachytherapy for BM treatment for “appropriately selected patients” and suggests placing patients on institutional registries or enrolling them in prospective studies to allow for more data to compare brachytherapy to techniques like SRS [54].

Although brachytherapy is designed to spare healthy brain tissue, the long-term cognitive effects of this treatment modality in patients with BM are not understood. In a 2016 prospective trial involving 24 patients with BM treated with surgical resection and Cs-131 seed placement, Pham et al. found a statistically significant improvement in cognitive functioning (measured by Mini Mental Status Exam scores) and quality of life (measured by the Functional Assessment of Cancer Therapy-Brain (FACT-BR) questionnaire) at both 4 and 6 months after brachytherapy treatment [20]. While there are studies investigating cognitive function and quality of life measures in patients treated with Cs-131 brachytherapy, they are too few to draw strong correlations between brachytherapy treatment and improved mental status.

Standardization of newer technologies, seed placement strategies, and dose calculations are necessary. Wernicke et al. initially proposed placement strategies for Cs-131 seeds [30] and the advent of GammaTile has further increased standardization [63,64]. However, more research and consensus guidelines could ensure consistent and optimized delivery of brachytherapy using various isotopes, particularly in the treatment of BMs. Additionally, more comparative studies evaluating the effectiveness, safety profile, and long-term outcomes associated with different isotopes are needed.

To date, there are two ongoing randomized controlled trials and one prospective cohort study analyzing the use of Cs-131 brachytherapy as an adjuvant therapy in the treatment of BMs (Table 3). Memorial Sloan Kettering Cancer Center is comparing the efficacy of surgical resection and Cs-131 versus surgical resection alone in maintaining freedom from local progression [65]. Two trials sponsored by GT Medical Technologies, Inc. are exploring the use of GammaTile. The first is assessing surgical resection plus GammaTile placement in various brain neoplasms including BM, with a primary outcome of surgical bed recurrence-free survival (NCT04427384). The second is comparing the intraoperative placement of GammaTile versus SRS 3–4 weeks after BM resection, with a primary outcome of surgical bed recurrence-free survival up to two years post-RT [66].

### Limitations

This scoping review is subject to several limitations. The nature of a scoping review constrains the depth of analysis typically conducted in systematic reviews or meta-analyses, as it aims to understand the breadth of the literature rather than evaluate the quality of the studies included. Additionally, by restricting the review to full-text articles published in English and focusing predominantly on adult populations, we may have omitted studies published in other languages or those involving pediatric subjects. Finally, the reliance on specific databases and search terms might have constrained the retrieval of all relevant studies, despite efforts to use a broad search strategy.

## 4. Conclusions

Advances in brachytherapy techniques for the treatment of brain metastases have shown significant promise in recent years. The ability to deliver targeted and localized radiation while sparing healthy brain tissue may lead to improved clinical outcomes and the potential for enhanced quality of life for patients. However, further research, including randomized controlled trials and longer-term follow-ups, is needed to establish the ideal role of brachytherapy in the management of brain metastases.

## Figures and Tables

**Table 1:** Overview of included published institutional series and case reports examining the use of brachytherapy in newly diagnosed BM treatment.

Study	Title	Journal	Country	Study Design	Cohort Size	Number of Patients with BM Treated with Brachytherapy	Primary Tumor	De-Novo or Recurrent BM	Single or Multiple BM	Isotope and Formulation	Outcomes
Bander, 2023 [8]	Safety and efficacy of Cesium-131 brachytherapy for brain tumors	Journal of Neuro-Oncology	USA	Retrospective	119	79	NSCLC, melanoma, breast, renal, GI, cervical, endometrial	De-Novo	Single	Cs-131 seeds	1-year survival rates were 53.3% (95%CI 41.9–64.6%) and 1-year local control rates were 84.7% for BM. Local control was superior in NSCLC relative to other BM pathologies (90.8% versus 76.5%). RN: 0
Bogart, 1999 [9]	Resection and permanent I-125 brachytherapy without whole brain irradiation for solitary brain metastasis from non-small cell lung carcinoma	Journal of Neuro-Oncology	USA	Retrospective	15	15	NSCLC	De-Novo	Single	I-125 seeds	Median survival: 14 months for all patients, 26 months for patients with single BM. No failures were seen with single BM < 2.5 cm. Recurrence rates both adjacent and outside the area of the initial brain lesion are similar to studies with resection + WBRT.
Brahimaj, 2016 [10]	Iodine-125 seed migration within brain parenchyma after brachytherapy for brain metastasis: case report	Journal of Neurosurgery	USA	Case report	1	1	Ovarian	De-Novo	Single	I-125 seeds	Seed migration along white matter tracts with neurological decline and death.
Dagnew, 2007 [11]	Management of newly diagnosed single brain metastasis using resection and permanent iodine-125 seeds without initial whole-brain radiotherapy: a two institution experience	Journal of Neurosurgery	USA	Retrospective	26	26	Lung, melanoma, colon, breast, renal, cervix, prostate, ovarian	De-Novo	Single	I-125 seeds	All patients treated with resection + I-125 seeds showed stable or improved KPS score. Median actuarial survival rate: 17.8 months.
Greenwald, 2019 [12]	Placement of 131Cs permanent brachytherapy seeds in a large combined cavity of two resected brain metastases in one setting: case report and technical note	Journal of Contemporary Brachytherapy	USA	Case report	1	1	Hypopharyngeal	De-Novo	Multiple	Cs-131 seeds	No recurrence; OS 20 months; no RN
Hirschfeld, 2016 [13]	Seed migration to the spinal canal after postresection brachytherapy to treat a large brain metastasis	Brachytherapy	USA	Case report	1	1	Esophageal	De-Novo	Single	Cs-131 seeds	Seed migration to the spinal canal with no new symptoms and no RN
Huang, 2009 [14]	Surgical resection and permanent iodine-125 brachytherapy for brain metastases	Journal of Neuro-Oncology	USA	Retrospective	40	40	Lung, breast, renal, melanoma	De-Novo	Both	I-125 seeds	Median OS: 11.3 months; 12.0 months in patients with de-novo BMs and 7.3 months in patients with recurrent BMs. 1-year local FFP rates for de-novo, recurrent, and all patients, respectively: 92%, 86%, and 88%; 1-year distant FFP rates: 29%, 43%, and 37%.
Julie, 2021 [15]	A matched-pair analysis of clinical outcomes after intracavitary cesium-131 brachytherapy versus stereotactic radiosurgery for resected brain metastases	Journal of Neurosurgery	USA	Retrospective	30	30	NSCLC, breast, CRC, GI, endometrial, melanoma, oropharynx, renal	De-Novo	Single	Cs-131 seeds	SD in LR: 10% for the resection + Cs-131 cohort and 28.3% for resection + SRS cohort (OR 0.281, 95% CI 0.082‚ 0.949; *p* = 0.049). NSD in RR, DR, and OS. Kaplan–Meier analysis showed a significantly higher likelihood of freedom from LR (*p* = 0.027) and DR (*p* = 0.018) after Cs-131 compared to SRS treatment (*p* = 0.027). NSD in likelihood of OS (*p* = 0.093). RN: 10% in SRS group and 3.3% in Cs-131 group (*p* = 0.417).
Mahase, 2017 [16]	Excellent Outcomes in a Geriatric Patient with Multiple Brain Metastases Undergoing Surgical Resection with Cesium-131 Implantation and Stereotactic Radiosurgery	Cureus	USA	Case report	1	1	Unknown	De-Novo	Multiple	Cs-131 seeds	No recurrence; OS 72 months; no RN
Nakaji, 2020 [17]	Resection and Surgically Targeted Radiation Therapy for the Treatment of Larger Recurrent or Newly Diagnosed Brain Metastasis: Results From a Prospective Trial	Cureus	USA	Prospective	11	11	Lung, breast	De-Novo and Recurrent	Both	Cs-131 GammaTile	1-year local FFP 91%; median OS 9.5 months (range, 1.4–28); RN 0%
Ostertag, 1995 [18]	Interstitial iodine-125 radiosurgery for cerebral metastases	British Journal of Neurosurgery	Germany	Retrospective	93	93	Lung, renal, melanoma, GI, GYN, breast, thyroid	De-Novo	Both	I-125 seeds	Median survival: 17 months in group IRT + percutaneous RT, 15 months in group IRT, 6 months in group IRT + prior RT. Favorable prognostic factors were a KPS > or = 70, single BM, absence of disseminated disease, and a time interval > 1 year between primary tumor and BM diagnosis.
Petr, 2009 [19]	Management of newly diagnosed single brain metastasis with surgical resection and permanent I-125 seeds without upfront whole brain radiotherapy	Journal of Neuro-Oncology	USA	Retrospective	72	72	Lung, breast, CRC, melanoma, ovarian, renal, prostate, thyroid, cervical, bladder	De-Novo	Single	I-125 seeds	Local control rate 93% at median 16 months. Distant brain failures occurred in 32%. RN: 5.6%. All patients had stable or improved KPS at 1 month. Median actuarial survival rate: 14 months; 2-year survival rate: 27%.
Pham, 2016 [20]	Neurocognitive function and quality of life in patients with newly diagnosed brain metastasis after treatment with intra-operative cesium-131 brachytherapy: a prospective trial	Journal of Neuro-Oncology	USA	Phase I/II Clinical Trial	24	24	Lung	De-Novo	Single	Cs-131 seeds	SD in MMSE and FACT-BR from baseline at 4 months (*p* = 0.001) and 6 months (*p* = 0.001) post-Cs131.
Raleigh, 2017 [21]	Resection and brain brachytherapy with permanent iodine-125 sources for brain metastasis	Journal of Neurosurgery	USA	Retrospective	95	95	Lung, melanoma, breast	De-Novo	Both	I-125 seeds	Crude local control: 90% at median 14.4-month follow-up. Median OS extended from 2.1 months in the shortest quartile to 62.3 months in the longest quartile (*p* < 0.0001). RN: 15%.
Rogers, 2006 [22]	Results of a phase II trial of the GliaSite radiation therapy system for the treatment of newly diagnosed, resected single brain metastases	Journal of Neurosurgery	USA	Prospective	71	71	NSCLC	De-Novo	Single	I-125 GliaSite	Local control rate: 82–87%. Median survival time and median duration of functional independence: 40 weeks. Results similar to resection + WBRT.
Romagna, 2016 [23]	Iodine-125 brachytherapy as upfront and salvage treatment for brain metastases: A comparative analysis	Strahlentherapie und Onkologie	Germany	Prospective	43	43	Lung, breast, GI, skin, prostate, renal, uterine, ovarian, MSK	De-Novo and Recurrent	Both	I-125 seeds	NSD in 1-year local/distant control rates after upfront and salvage SBT (94%/65% vs. 87%/57%, *p* = 0.45, respectively). Grade I/II toxicity seen in 2 patients after salvage SBT.
Ruge, Kocher, 2011 [24]	Comparison of stereotactic brachytherapy (125 iodine seeds) with stereotactic radiosurgery (LINAC) for the treatment of singular cerebral metastases	Strahlentherapie und Onkologie	Germany	Retrospective	219	77	NSCLC, breast, melanoma, renal, CRC	De-Novo	Single	I-125 seeds	NSD in median OS or actuarial local/distant control at 1 year. No permanent grade III/IV CNS toxicity.
Ruge, Suchorska, 2011 [25]	Stereotactic 125iodine brachytherapy for the treatment of singular brain metastases: closing a gap?	Neurosurgery	Germany	Retrospective	90	90	NSCLC, breast, renal, melanoma, CRC	De-Novo	Single	I-125 seeds	Median OS: 8.5 months. 1-year actuarial local and distant recurrence: 5.4% and 46.4%, respectively. KPS ≥ 70 (*p* < 0.002), stable systemic disease (*p* < 0.02), and a prolonged (>12 month) interval between diagnosis and brachytherapy (*p* < 0.05) significantly improved survival.
Schulder, 1997 [26]	Permanent low-activity iodine-125 implants for cerebral metastases	Journal of Neuro-Oncology	USA	Retrospective	13	13	Lung, germ cell, breast, melanoma, renal	De-Novo	Single	I-125 seeds	All patients received WBRT. Implant dose ranged from 43 Gy–132 Gy, mean 83 Gy. Survival after implantation ranged from 2 weeks–9 years, median 9 months.
Warren, 2021 [27]	Surgical Outcomes of Novel Collagen Tile Cesium Brachytherapy for Recurrent Intracranial Tumors at a Tertiary Referral Center	Cureus	USA	Retrospective	12	2	Lung, breast	De-Novo	Single	Cs-131 GammaTile	Loco-regional FFP 58.3%; median OS 7 months (IQR, 3–10); RN 8.3%
Wernicke, 2014 [28]	Phase I/II study of resection and intraoperative cesium-131 radioisotope brachytherapy in patients with newly diagnosed brain metastases	Journal of Neurosurgery	USA	Phase I/II Clinical Trial	24	24	Lung, breast, renal, melanoma, colon, cervix	De-Novo	Single	Cs-131 seeds	1-year FFP: local 100%, regional 93.8%, distant 48.4%; 1-year OS 50%; RN 0%
Wernicke, Hirschfeld, 2017 [29]	Clinical Outcomes of Large Brain Metastases Treated With Neurosurgical Resection and Intraoperative Cesium-131 Brachytherapy: Results of a Prospective Trial	International Journal of Radiation Oncology Biology Physics	USA	Prospective	46	46	Lung, colon, breast, melanoma, uterine, esophageal, renal, hepatobiliary, tonsillar	De-Novo	Both	Cs-131 seeds	1-year FFP: local 100%, regional 89%, distant 52%; 1-year OS 58%; RN 0%
Wernicke, Lazow, 2016 [30]	Surgical Technique and Clinically Relevant Resection Cavity Dynamics Following Implantation of Cesium-131 (Cs-131) Brachytherapy in Patients With Brain Metastases	Operative Neurosurgery	USA	Phase I/II Clinical Trial	24	24	Lung, breast, melanoma, uterine, renal, CRC, esophageal	De-Novo	Single	Cs-131 seeds	SD in post-implantation cavity shrinkage resection + Cs-131 56.5% vs. resection + SRS 84.8% (*p* = 0.008)
Wernicke, Yondorf, 2016 [28]	The cost-effectiveness of surgical resection and cesium-131 intraoperative brachytherapy versus surgical resection and stereotactic radiosurgery in the treatment of metastatic brain tumors	Journal of Neuro-Oncology	USA	Phase I/II Clinical Trial	24	24	Lung, breast, colon	De-Novo	Single	Cs-131 seeds	Direct costs per patient: resection + Cs-131 USD 19,271 vs. resection + SRS USD 44,219; 1-year OS: resection + SRS 61% vs. resection + Cs-131 50% (*p* = 0.137); QALY compared (*p* < 0.0001); ICER resection + SRS significantly inferior (*p* < 0.0001); resection + Cs-131 significantly more cost-effective.
Xia, 2018 [31]	Outcomes of Metastatic Brain Lesions Treated with Radioactive Cs-131 Seeds after Surgery: Experience from One Institution	Cureus	USA	Retrospective	9	9	Breast, lung, melanoma, uterine, thyroid, CRC	De-Novo	Both	Cs-131 seeds	1-year FFP: local 100%, distant 66.7%; median OS 9.4 months (range, 1.3–42.2); RN 0%
Yang, 2022 [32]	Iodine-125 brachytherapy treatment for newly diagnosed brain metastasis in non-small cell lung cancer: A biocentric analysis	Frontiers of Oncology	China	Retrospective	99	59	NSCLC	De-Novo	Single	I-125 seeds	SD in 6-month PFS rate in I-125 group vs. EBRT group (*p* = 0.002). NSD in 12-month PFS rate (*p* = 0.184). NSD in 12- (*p* = 0.839) and 24-month (*p* = 0.284) OS. NSD in median OS (*p* = 0.525) or PFS (*p* = 0.425).
Yondorf, 2020 [33]	Dosimetric differences between cesium-131 and iodine-125 brachytherapy for the treatment of resected brain metastases	Journal of Contemporary Brachytherapy	USA	Phase I/II Clinical Trial	24	24	Lung, breast, renal, melanoma, colon, cervix	De-Novo	Single	Cs-131 seeds	Dosimetric characteristics of Cs-131 compared to I-125 implants: Cs-131 exposes lower volumes of brain tissue to radiation, provides more uniform dosing, and targets the desired CTV volume more accurately.

BM—brain metastasis; CI—confidence interval; Cs-131—Cesium-131 brachytherapy; CTV—Clinically targeted volume; DR—distant recurrence; EBRT—external beam radiotherapy; FACT-BR—functional assessment of cancer therapy—brain; FFP—freedom from progression; ICER—incremental cost-effectiveness ratios; I-125—Iodine-125 brachytherapy; IQR—interquartile range; IRT—interstitial radiotherapy; KPS—Karnofsky performance status; LR—local recurrence; MMSE—mini-mental status examination; NSD—no significant difference; OR—odds ratio; OS—overall survival; QALY—quality-adjusted life years; RN—radiation necrosis; RR—regional recurrence; RT—radiotherapy; SBT—stereotactic brachytherapy; SD—significant difference; SRS—stereotactic radiosurgery; WBRT—whole-brain radiotherapy.

**Table 2:** Overview of included published institutional series and case reports examining the use of brachytherapy in recurrent BM treatment.

Study	Title	Journal	Country	Study Design	Cohort Size	Number of Patients with BM Treated with Brachytherapy	Primary Tumor	De-Novo or Recurrent BM	Single or Multiple BM	Isotope and Formulation	Outcomes
Bernstein, 1995 [34]	Brachytherapy for recurrent single brain metastasis	Canadian Journal of Neurological Sciences	Canada	Retrospective	10	10	Lung, breast	Recurrent	Single	I-125 seeds	1-year FFP: local 100%, regional 93.8%, distant 48.4%. 1-year OS 50%. RN 0%
Chen, 2022 [35]	Resection with intraoperative cesium-131 brachytherapy as salvage therapy for recurrent brain tumors	Journal of Neurosurgery	USA	Retrospective	36	20	Lung, breast, GI	Recurrent	Both	Cs-131 GammaTile	Local FFP 90.5%; regional FFP 90.5%; distant FFP 47.6%; median OS 26.7 months (range, 15.6–36.7); RN 11.9%
Cummins, 2022 [36]	Salvage Surgery for Local Control of Brain Metastases After Previous Stereotactic Radiosurgery: A Single-Center Series	World Neurosurgery	USA	Retrospective	43	43	Melanoma, NSCLC, breast, GI, renal	Recurrent	Both	Cs-131 seeds and I-125 seeds	Brachytherapy was associated with improved local control (HR, 0.15; 95% CI, 0.04–0.6; *p* = 0.008). For patients treated with SRS before salvage surgery, both brachytherapy (HR, 0.07; 95% CI, 0.01–0.39; *p* = 0.002) and postoperative adjuvant SRS (HR, 0.14; 95% CI, 0.02–1.00; *p* = 0.05) were associated with improved local control compared with no adjuvant radiation therapy.
Dharnipragada, 2023 [37]	GammaTile (GT) as a brachytherapy platform for rapidly growing brain metastasis	Neuro-Oncology Advances	USA	Retrospective	10	10	Lung, breast, melanoma, uterine	Recurrent	Single	Cs-131 GammaTile	40% of patients showed symptomatic improvement. Remaining 60% showed stable neurologic conditions.
Nakaji, 2020 [17]	Resection and Surgically Targeted Radiation Therapy for the Treatment of Larger Recurrent or Newly Diagnosed Brain Metastasis: Results From a Prospective Trial	Cureus	USA	Prospective	11	11	Lung, breast	De-Novo and Recurrent	Both	Cs-131 GammaTile	1-year local FFP 91%; median OS 9.5 months (range, 1.4–28); RN 0%
Romagna, 2016 [23]	Iodine-125 brachytherapy as upfront and salvage treatment for brain metastases: A comparative analysis	Strahlentherapie und Onkologie	Germany	Prospective	43	43	Lung, breast, GI, skin, prostate, renal, uterine, ovarian, MSK	De-Novo and Recurrent	Both	I-125 seeds	NSD in 1-year local/distant control rates after upfront and salvage SBT (94%/65% vs. 87%/57%, *p* = 0.45, respectively). Grade I/II toxicity seen in 2 patients after salvage SBT.
Ruge, Kickingereder, 2011 [38]	Stereotactic biopsy combined with stereotactic (125) iodine brachytherapy for diagnosis and treatment of locally recurrent single brain metastases	Journal of Neuro-Oncology	Germany	Retrospective	30	30	Breast, NSCLC, melanoma, CRC, renal, esophageal	Recurrent	Single	I-125 seeds	27 treated with SBT for recurrent BM. Median survival: 14.8 months. 1-year actuarial local and distant relapse: 6.7% and 45.5%, respectively. No grade III/IV CNS toxicity, even among tumors > 30 mm.
Wernicke, Smith, 2017 [39]	Cesium-131 brachytherapy for recurrent brain metastases: durable salvage treatment for previously irradiated metastatic disease	Journal of Neurosurgery	USA	Retrospective	13	13	Lung, breast, melanoma, pancreatic, gastric	Recurrent	Both	Cs-131 seeds	1-year FFP: local 83.3%, regional 55.6%, distant 46.7%; 1-year OS 24.7%; RN 6.7%
Wu, 2022 [40]	Salvage brachytherapy for multiply recurrent metastatic brain tumors: A matched case analysis	Neuro-Oncology Advances	USA	Retrospective	14	14	Lung, breast, melanoma	Recurrent	Both	Cs-131 seeds and I-125 seeds	SD in FFLR compared to prior treatments (median 7.39 vs. 5.51 months, *p* = 0.011) for multiply recurrent BMs. Compared to an independent matched cohort, brachytherapy demonstrated superior FFLR (median 8.49 vs. 1.61 months, *p* = 0.004) and longer median OS (11.07 vs. 5.93 months, *p* = 0.055).

BM—brain metastasis; CI—confidence interval; Cs-131—Cesium-131 brachytherapy; EBRT—external beam radiotherapy; FFP—freedom from progression; HR—hazard ratio; I-125—Iodine-125 brachytherapy; KPS—Karnofsky performance status; NSD—no significant difference;; OS—overall survival; RN—radiation necrosis; RT—radiotherapy; SBT—stereotactic brachytherapy; SD—significant difference; SRS—stereotactic radiosurgery.

**Table 3:** Overview of ongoing clinical trials evaluating the use of brachytherapy in the treatment of BM.

NCT Number	Study Title	Study Sponsor	Study Design	Conditions	Summary	Interventions	Primary Outcomes
04690348	Intracavitary Carrier-Embedded Cs-131 Brachytherapy for Recurrent Brain Metastases: A Randomized Phase II Study	Memorial Sloan Kettering Cancer Center	RCT	Recurrent BM	Resection + Cs-131 vs. resection only	PROCEDURE: Craniotomy RADIATION: Cs-131 brachytherapy	FFLP
04427384	A Multicenter Observational Study of GammaTile Surgically Targeted Radiation Therapy (STaRT) in Intracranial Brain Neoplasms	GT Medical Technologies, Inc.	Prospective Cohort	Primary and Secondary Brain Tumors	Evaluate real-world clinical outcomes and patient-reported outcomes that measure the effectiveness and safety of STaRT post-resection.	DEVICE: Gamma Tile-Surgically Targeted Radiation Therapy (STaRT)	Surgical bed recurrence-free survival
04365374	Post-Surgical Stereotactic Radiotherapy Versus GammaTile-ROADS (Radiation One and Done Study)	GT Medical Technologies, Inc.	RCT	BM	This trial will be a randomized controlled study comparing the efficacy and safety of intraoperative radiation therapy using GammaTile versus SRS 3–4 weeks following metastatic tumor resection, which is the current standard of care.	DEVICE: Gamma Tile-Surgically Targeted Radiation Therapy (STaRT) RADIATION: Stereotactic Radiation Therapy	Surgical bed recurrence-free survival from the time of randomization up to 2 years post-radiation.

BM—brain metastasis; Cs-131—cesium-131 brachytherapy; FFLP—freedom from local progression; NCT—national clinical trial; RCT—randomized controlled trial; SRS—stereotactic radiosurgery.
